# Manipulation complexity in primates coevolved with brain size and terrestriality

**DOI:** 10.1038/srep24528

**Published:** 2016-04-14

**Authors:** Sandra A. Heldstab, Zaida K. Kosonen, Sonja E. Koski, Judith M. Burkart, Carel P. van Schaik, Karin Isler

**Affiliations:** 1 Department of Anthropology, University of Zurich, Winterthurerstrasse 190, 8057 Zurich, Switzerland; 2 University of Helsinki, Centre of Excellence in Intersubjectivity in Interaction. P.O.Box 4, Vuorikatu 3, 00014 Helsinki, Finland

## Abstract

Humans occupy by far the most complex foraging niche of all mammals, built around sophisticated technology, and at the same time exhibit unusually large brains. To examine the evolutionary processes underlying these features, we investigated how manipulation complexity is related to brain size, cognitive test performance, terrestriality, and diet quality in a sample of 36 non-human primate species. We categorized manipulation bouts in food-related contexts into unimanual and bimanual actions, and asynchronous or synchronous hand and finger use, and established levels of manipulative complexity using Guttman scaling. Manipulation categories followed a cumulative ranking. They were particularly high in species that use cognitively challenging food acquisition techniques, such as extractive foraging and tool use. Manipulation complexity was also consistently positively correlated with brain size and cognitive test performance. Terrestriality had a positive effect on this relationship, but diet quality did not affect it. Unlike a previous study on carnivores, we found that, among primates, brain size and complex manipulations to acquire food underwent correlated evolution, which may have been influenced by terrestriality. Accordingly, our results support the idea of an evolutionary feedback loop between manipulation complexity and cognition in the human lineage, which may have been enhanced by increasingly terrestrial habits.

Humans stand out among animals in having both a very complex foraging niche, built around sophisticated technology, and an unusually large brain. Is this combination just a coincidence, or instead the product of correlated evolution, as suggested by the relationship between use and manufacture of tools and brain size^1^,^2^? In carnivores, no correlation between brain size and forelimb dexterity during feeding was found^3^. Here, using a new method of assessing manipulation complexity in food-related contexts, we examine the relationship between foraging niche complexity and brain size for 36 non-human primate species from various taxonomic groups.

Manipulation complexity was previously defined according to a variety of contrasts: (i) the use of one hand rather than two hands in bimanual coordination; (ii) asymmetrical bimanual manipulation (i.e., both hands simultaneously performing different actions) versus symmetrical bimanual manipulation (i.e., both hands simultaneously performing the same action); (iii) uncoordinated two-handed patterns (i.e. both hands performing actions, independently) versus coordinated two-handed patterns (i.e. both hands performing actions dependent on each other in space and/or time); or (iv) any combination of these criteria^4^,^5^,^6^,^7^,^8^. However, in all these reports on how primates use their hands to perform object manipulation, no explicit evaluation of the level of complexity was undertaken. Therefore, our first aim was to test whether distinct food manipulation categories can be ranked cumulatively across species according to their difficulty. We can speak of “manipulation complexity” if there is a clear ranking pattern, that is if species which are able to perform a given type of manipulation are also able to perform all manipulations of a lower rank. Such an empirical evaluation of complexity, without a priori assumptions of which manipulations are more complex than others, has to our knowledge not yet been undertaken.

Second, we tested whether a species’ manipulation complexity is related to cognitively challenging food acquisition techniques, such as, extractive foraging or tool use^2^,^9^. Effective extractive foraging is likely to require complex manipulative skills, because finely tuned movements are an advantage for removing and holding food or manipulating it with different objects. Tool use, on the other hand, is considered cognitively and manipulatively difficult as it often involves bimanually coordinated actions causally relating two or more external objects^10^.

Third, an emerging consensus is that foraging skills have played an important role in cognitive evolution^1^,^2^,^4^,^11^,^12^. We therefore also tested whether the degree of food manipulation complexity is related to brain size or cognitive test performance.

We further tested the influence of other factors that may have affected the correlated evolution between brain size and manipulation complexity: diet quality and terrestriality^13^. High-quality diets may require more complex motor and cognitive skills than low-quality diets. For example, insectivorous and frugivorous primates may need to perform more complex manual food processing than folivorous primates, and are more likely to use tools^12^. Likewise, in a terrestrial habitat, in contrast to arboreal contexts, hands are less commonly needed for positional support, which may allow for the evolution of morphologies capable of more actions on objects and the use of complex actions that require the coordinated involvement of both hands^14^. In addition, discarded tools remain close to where they are used in terrestrial settings, while they tend to disappear from sight after being dropped in arboreal ones^15^,^16^.

If manipulation complexity and brain size coevolved among primates, and thus ecological requirements partly drove brain size evolution in this lineage, it is interesting to ask whether the human manipulation complexity score fits into the pattern based on the relationship between manipulation complexity and brain size in nonhuman primates. If humans fit the primate trend, this suggests that the process of correlated evolution between brain size and foraging ecology may also have played a major role in human brain evolution.

## Material and Methods

### Data collection

We assessed manipulations in food contexts in captive individuals of 36 primate species (Supplementary Table S1). All subjects were housed in their home enclosures in single-species groups of 2 to 27 individuals that included at least one adult male and one adult female, except for *Saimiri sciureus* (seven males), *Propithecus verreauxi* (two males, each housed together with a *Eulemur mongoz*) and *Leontopithecus rosalia* (one male). We sampled all available individuals, but immatures were excluded because their manipulation patterns are usually qualitatively different from those of adults^14^,^15^. Data on human manipulation complexity was collected in a similar way, observing everyday food consumption in a “natural” setting (in the cafeteria of the University of Zurich, Switzerland).

Data were collected by behavioural sampling between October 2011 and February 2014, for a total of 112 hours in various zoos (Supplementary Table S1). Manipulation was defined as making physical contact with a food item with the forelimbs, and thus did not include visual exploration or sniffing without contact. Behavioural sampling was conducted in bouts; a bout started as soon as an individual started to manipulate a food item and ended when contact was terminated or after a maximal duration of 5 minutes. After each individual bout, there was an interval of at least 2 minutes without sampling. At least 10 bouts per sex, and thus 20 bouts per species, were collected for most species (Supplementary Table S1). In total, 962 bouts were recorded.

**Figure 1:**
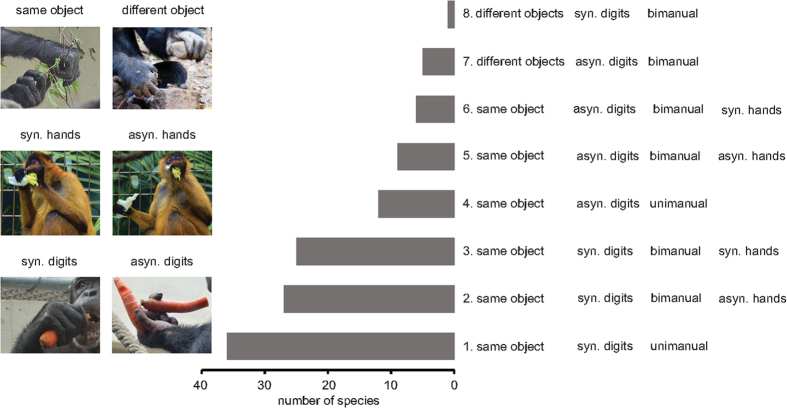
The eight manipulation complexity categories found through the Guttman scaling method (increasing complexity from category 1 to category 8), and the number of species able to perform actions in a particular manipulation complexity category. Copyright S.A. Heldstab, *Pan troglodytes* nut cracking K. Koops.

Observed manipulation bouts were divided into eight categories. These categories were based on all possible combination of the following: (i) use of the forelimbs, subdivided into unimanual and bimanual actions, (ii) asynchronous and synchronous use of hands, and (iii) asynchronous and synchronous use of digits. Furthermore, in bimanual actions we distinguished between the hands manipulating the same object or different objects, e.g. whether hands were both manipulating a fruit (same object) versus one hand is manipulating with a stick and the other hand is holding the fruit (different object) (Fig. 1). We only scored the presence of manipulation categories if the observed individuals of a primate species performed a manipulation category at least twice. Frequency or duration of manipulation categories were not assessed.

Agreement between the two observers (inter-observer reliability) was assessed with the kappa statistic^17^, which corrects for agreement due to chance and was applied to the entire coding scheme. The acceptance criterion was set at 0.70, and all kappa statistics were substantially above this basal criterion (manipulation observed: *K* = 0.85, *n* = 20; level of manipulation: *K* = 0.72, *n* = 18; context of manipulation: *K* = 0.94, *n* = 18). Moreover, each species was observed by both observers together for at least 10 minutes to ensure reliability between different species.

### Complexity levels

Using the deterministic Guttman scaling method based on the description of Green^18^, we assessed whether the manipulation categories followed a cumulative ranking. Using Guttman’s scaling method^19^ we can derive a rank order in a given set of skills, yielding a difficulty scale that is as cumulative as possible. The manipulation skills are ranked such that if an individual is able to perform a particular skill *N*, then that individual must also be able to perform all or most easier skills <*N*. Thus, in the ideal case of perfect nesting, if an individual’s score is known, this predicts the individual’s performances in all skills in the scale. For any empirical set of observed skills, the coefficient of reproducibility^19^ indicates to which extent the skills indeed do fit such a cumulative scale, or, in other words, whether there is a level of difficulty or complexity of the respective skills.

After ranking complexity levels of manipulations using the Guttman scale using all data on all species, mean manipulation complexities were calculated for each species as follows: For each manipulation bout the highest rank reached during the bout was determined. The mean complexity score for each species was then averaged over all observed bouts.

Additionally, we also performed all analyses using the highest manipulation complexity score reached over all observation bouts for each species. The results using this highest manipulation complexity score are largely identical to those obtained with mean complexity scores, but there is a ceiling effect and thus fewer distinctions between species (Supplementary Table S5).

### Cognitive test performance and brain size

Two different meta-analyses of cognitive performance of a broad set of primate species provide quantitative estimates of cognitive test performance across primate genera^11^,^20^. Using the mean manipulation complexity for each genus (*n* = 15 genera for^20^/*n* = 19 genera for^11^), this allows for a direct test of a relationship between manipulation complexity and cognitive test performance on the genus level.

Endocranial volumes of mostly wild-derived female primates^21^ were used as a proxy of brain size on the species level (*n* = 36). To remove allometric effects of body size, female body mass was integrated as an independent variable in all multiple regression analyses^22^. Sources of data on endocranial volumes and body mass are given in Supplementary Table S1. Results showing that brain size is related to cognitive abilities in our primate sample are shown in Supplementary Table S4.

To test whether manipulative skills are particularly high in species known to perform cognitively challenging food acquisition techniques, tool use and extractive foraging were coded as binary variables (present = 1, absent = 0) for each species with data from the literature from wild primates^23^,^24^,^25^,^26^. In addition, we analysed correlations of manipulation complexity with neocortex and cerebellum size and with foraging group size as a measure of social complexity (results are reported and discussed in detail in the Supplementary material).

To test whether the human manipulation complexity score fits into the pattern between manipulation complexity and brain size in nonhuman primates, we conducted a bootstrapping analysis. The mean and width of the 95% confidence intervals were generated for sample estimates by bootstrapping (1000 iterations)^27^. At each iteration, a bootstrap sample of 25 and 30 nonhuman primate species was constructed by sampling at random without replacement. In a next step we tested whether the estimate of the phylogenetic generalized least-squares regression including the human manipulation complexity score was within the confidence limits calculated on the basis of nonhuman primates. If the data point for humans lies outside the confidence limits, then humans can be considered significantly different from the nonhuman primate trend with *P* < 0.05 (two-tailed). Furthermore, by using the “predict()” function in the “caper” package^28^ we calculated the human manipulation score which would be predicted for its brain size on the basis of the relationship between manipulation complexity and brain size in nonhuman primates.

### Diet quality and terrestriality

To test whether diet quality is related to manipulation complexity, we integrated diet quality data from wild primates^24^ (and references therein). Diet quality was determined by using temporal variation in the time spent feeding on diet components (and thus their estimated consumption) as in van Woerden^29^: Monthly mean intake of each food category, as estimated by feeding time, was multiplied by its relative energetic quality (8 for insects; 5 for fruits, seeds, and flowers; 3 for gum and young leaves; and 1 for mature leaves, as calculated from g crude fibre/kg dry matter by Langer^30^). Fibre content is commonly used as a measure of digestibility and thus energy gained per unit time^31^. An alternative, categorical scheme of main diet categories was defined as follows: Insectivorous and frugivorous primates are coded as 1 (diets requiring complex manipulation), and folivorous and gummivorous primates are coded as 0 (diets requiring less complex manipulation) (Supplementary Table S1). Results using this alternative, categorical scheme of main diet categories are largely identical to those obtained with diet quality, and diet categories are highly correlated because diets related to complex manipulations, such as fruits and insects, also have higher nutritional values (Supplementary Table S8).

As a proxy of terrestriality, primate species were placed in one of three categories based on their main travel habit, as follows: (1) terrestrial (more than 60% terrestrial), (0.5) partly terrestrial (more than 20% terrestrial) and (0) arboreal. Data on terrestriality were taken from the published literature^23^,^24^.

### Statistical analyses

All statistical analyses were performed using JMPTM 10.0^32^ and R2.13.1^33^. The method of phylogenetic generalized least-squares regression (PGLS)^34^ with the “caper” package^28^ was used to control for phylogenetic non-independence. Phylogeny was based on a composite supertree including branch length estimations^35^ (Supplementary Fig. S2). Results using an alternative phylogenetic tree (phylogeny based on version 3 of 10K trees^36^) remained largely similar (Supplementary Table S9). The values of body mass, brain size and diet quality were log_e_ transformed in order to reach residuals evenly distributed around zero.

Manipulation complexity of species that use tools or do not use tools as well as of species with or without extractive foraging was compared using PGLS. The correlation of cognitive test performance scores, brain size, diet quality, and terrestriality with manipulation complexity was tested for each variable separately. In a second step, a multiple regression model was run to include manipulation complexity as the response, brain size as effect, and diet quality, terrestriality and body mass as covariates. As diet quality data was not available for *Ateles fusciceps* and *Saguinus imperator*, those two species were excluded from the multiple regression models, yielding a sample size of *n* = 34 non-human primate species. To choose the best fitting from a set of models, the AIC values (Akaike Information Criterion^37^) of different models were compared. In these models, we also tested for interaction effects between the predictor variables. Bivariate plots of manipulation complexity against brain size, cognitive test performance, diet quality residuals (corrected for body mass), and terrestriality are shown for illustrative purpose only.

### Ethical statement animals

All the observations were carried out in accordance with the Swiss legislation on animal experimentation and formally approved by the Kantonales Veterinäramt of Zurich.

### Ethical statement humans

All the observation were carried out in accordance with the Swiss legislation on research involving human subjects. The subjects provided written informed consent, and the observations were approved by the Ethikkommission für psychologische und verwandte Forschung of the Philosophische Fakultät der Universität Zürich (step 1).

## Results

We found that manipulation categories follow a cumulative ranking (Fig. 1). In total, 86% of species’ performances exactly fitted the resulting Guttman scale, and the coefficient of reproducibility was close to 1 (0.92), indicating that manipulation complexity is indeed cumulative across species. For example, species that are able to perform category 4 mostly also exhibit categories 1, 2, and 3, therefore category 4 can be seen as more complex than the latter.

The scale implies the following scale of manipulative complexity: First, manipulating two objects simultaneously is more complex than manipulating only one object with both hands. Second, the complexity of a manipulation increased with the capability to move digits asynchronously, and when using both hands instead of just one hand for the action. Third, manipulations with synchronous hand use tended to be more complex than manipulations with asynchronous hand use.

**Figure 2:**
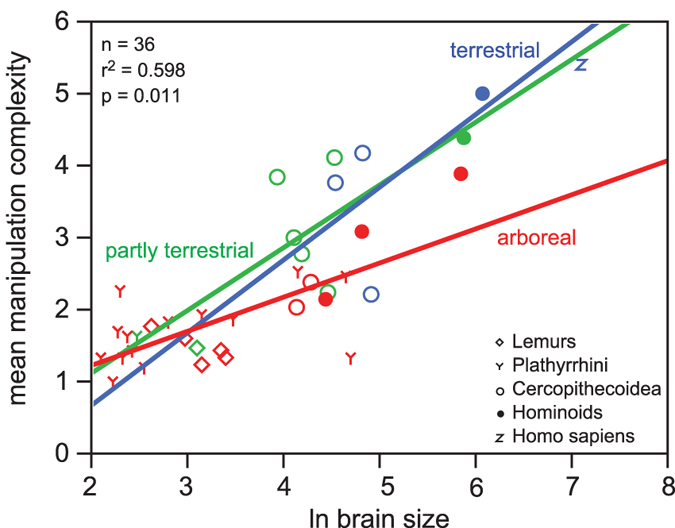
Relationship between manipulation complexity and ln (brain size), for various types of substrate use (raw species values, blue = terrestrial, green = partly terrestrial, red = arboreal) shown for visualisation purpose. Species values are listed in Supplementary Table S1. *Homo sapiens* is not included in the calculation of the correlation and is only shown for illustrative purposes. Statistics see Table 2.

Manipulation complexity was significantly higher (*P* = 0.020) in primate species that regularly use tools and substantially higher (*P* = 0.056) in species that exhibit extractive foraging (Table 1, Supplementary Fig. S2). Manipulation complexity was also positively correlated with relative brain size (Table 2, Fig. 2) and with performance on cognitive tests (Table 1, Supplementary Fig. S3).

From visual inspection, the relationship between manipulation complexity and brain size appeared to be steeper in terrestrial species, but the effect of the interaction was not statistically significant (Table 2 and Supplementary Table S2, Fig. 2 and Supplementary Fig. S3). Diet quality did not affect the relationship between manipulation complexity and brain size (Table 2 and Supplementary Fig. S3).

Humans reached by far the highest manipulation complexity score of all tested species (Supplementary Table S1). Including *Homo sapiens* in the analyses increased the magnitude and significance of the difference in manipulation complexity between species that use tools (*P* = 0.006) and perform extractive foraging (*P* = 0.048), compared to species that do not (Table 2 and Supplementary Table S3). Furthermore, the human data point always lies above the upper confidence limit calculated using the relationship between manipulation complexity and brain size of nonhuman primates. This indicates that the human manipulation complexity score and brain size were higher compared to nonhuman primates (Table 3). The human manipulation complexity score calculated using the bootstrapping approach, on the basis of nonhuman primates, was 4.98, which is below the actual measured score of 5.40. Therefore, the human manipulation complexity score was higher than predicted for our brain size, and additional factors (e.g. bipedality, see discussion) may have enhanced human manipulative skills.

## Discussion

If we aim to understand the evolutionary link between primate manipulative skills and the flourishing of complex technology among humans, we need explicit and consistent operational definitions of manipulative skills as well as a thorough evaluation of the levels of difficulty of the various manipulative actions across a broad variety of primate species^38^. Here, we provide manipulation categories that can be easily distinguished, and show that they can be ranked cumulatively according to their complexity across a broad range of primate species, and that this complexity measure is correlated with brain size and relevant ecological aspects (extractive foraging and tool use).

Consistent with previous studies, non-human primate taxa considered to be the most dexterous–specifically chimpanzees^16^,^39^,^40^, gorillas^4^,^41^, orangutans^4^,^16^,^42^, geladas^4^ and macaques^4^,^7^–also showed high manipulative complexity in our study. These findings support the accuracy of our rankings of the manipulation categories according to difficulty.

Furthermore, the different manipulation categories found in this study correspond quite well to the food processing behaviours observed in wild living primates. For instance, the 72 functionally distinct manipulative actions in wild living mountain gorillas recorded by Byrne and colleagues^41^ would be classified as levels 1 to 6 in our manipulation complexity scheme. But we also observed tool use of level 7 in the captive Western lowland gorillas. Chimpanzees of the Taï National Park (Côte d’Ivoire)^43^ and of the Mahale Mountains National Park (Tanzania)^44^ are able to perform all manipulation categories 1 to 7 found in our study.

Free ranging Japanese macaques^7^ exhibit asynchronous and synchronous hand and digit use according to complexity levels 1–6 in our scheme, matching our findings for Tonkean macaque and Barbary macaque. The long-tailed macaques, on the other hand, for which we also found level 7 manipulations, are renowned for high manipulation complexity, tool use and extractive foraging in the wild^4^.

### Complexity of manipulations

Most previous studies on object manipulation and tool-use in primates were either experimentally induced or directed towards experimentally provided standardized objects (e.g.^5^,^39^,^40^). In contrast, in our study, manipulations were unconstrained and undertaken in a non-experimental setting, without introducing new objects or changes to the daily routine of the animals. Animals were free to select food items and any additional objects available within the enclosure. Our data demonstrate that the types of food (sometimes, including chopped fruits or pellets) available to the primates under study did not limit their capacity for manipulations. First, several species exhibited manipulations of high complexity classified in category 7. Second, even species with generally low manipulation complexity showed a few manipulations of higher complexity. Third, species differed in manipulation complexity even when the food items were identical. For example, all primates in the Parc Zoologique et Botanique de Mulhouse (France) were fed with similar-sized chopped fruits. Yet the highest manipulation complexity category ever reached, by different species held in this specific zoo, varied between 1 and 6. In summary, manipulations were not constrained by the availability of a particular food item or object, and all species had the opportunity to show the full range of manipulations.

In our study, the complexity of a manipulation increased when using both hands instead of just one hand for the action. Other studies have indeed found that patterns performed unimanually are more straightforward for the brain to program than patterns performed bimanually^5^,^6^,^8^. For instance, reaching, grasping or holding a food item arise very early in infancy, suggesting a low level of manipulative complexity^45^.

Second, we found that manipulating two objects is more complex than manipulating the same object with both forelimbs. This corresponds to earlier studies on ontogeny of nut cracking in wild chimpanzees. Biro and colleagues^46^ found that in infants in early stages of development interactions with nuts or stones were restricted to the manipulation of single objects on their own, such as holding a stone or rolling a nut. This stage was then followed at later ages by performing actions on multiple objects, indicating that manipulating one object is less complex than manipulating two objects.

Third, the complexity of a manipulation increased with the capability to move digits asynchronously. Again, this pattern also occurs during ontogeny. During human development, individuated movements of the fingers become superimposed on more fundamental grasping movements involving synchronous digits. Reflexive closure of the entire hand, which is present at birth, is followed by voluntary grasping at 2–3 months of age. Thumb opposition and finger individuation start to appear at 10–12 months indicating that manipulations with asynchronous digits are more complex than with synchronous digits^45^. Furthermore, a study in capuchins showed that they performed manipulation involving synchronous digits much quicker than manipulations involving asynchronous digit use suggesting that using the digits asynchronously seems to be more complex than synchronous digit use^47^.

Fourth, manipulations with synchronous hand use tended to be more complex than manipulations with asynchronous hand use. This finding is surprising as previous studies suggested that patterns of asynchronous hand use are more complex than those of synchronous hand use within species^5^,^6^,^8^. In future studies we will revisit this unexpected result by assessing whether the order of emergence of these manipulation categories during ontogeny matches the order of the complexity scale found in this study.

### Ecology

Primate species engaging in extractive foraging and tool use tended to have higher manipulation complexity than those that do not. Previous studies have shown that exactly these two food acquisition modes are mastered relatively late in development, and attributed their late appearance to them being cognitively demanding and involving complex manipulative patterns^15^,^42^. Both suggestions are supported by our results.

### Cognitive abilities

Contrary to a study on forelimb dexterity in carnivores^3^, we found that in primates brain size and cognitive test performance exhibit correlated evolution with manipulation complexity. This relationship between brain size and manipulation complexity persists even after controlling for foraging group size (Supplementary Table S7). The difference between primates and carnivores may be due to the underlying adaptation for grasping in the primate forelimb. Correlated evolution of brain size and manipulation complexity may be hindered by a phylogenetic constraint of paw morphology in carnivores, a group in which arboreality is only a secondary adaptation. To explain patterns of correlated evolution that shaped brain size variation across mammals, such discrepancies between orders should be studied in more detail.

Additional results on the relationship between the size of specific brain regions (neocortex or cerebellum size) and manipulation complexity showed that relative cerebellum size was not correlated with manipulation complexity (Supplementary Table S6). Relative neocortex size on the other hand was positively correlated with manipulation complexity (Supplementary Table S6). This may indicate a closer link between manipulation complexity and cognitive rather than motor skills. However, the cerebellum is involved not only in sensory-motor control and automatized learning of motor skills, but may also play a role in understanding and producing complex behavioural sequences including tool use^1^,^48^. Because our results on brain parts depend on a relatively small sample, they must be regarded with caution.

### Terrestriality

The primate pattern suggests that not only full terrestriality, but already a partly terrestrial habit may have positively affected the correlated evolution between manipulation complexity and brain size. The secondary adoption of a partly terrestrial habit may therefore have facilitated innovation by allowing more frequent and repeated actions on objects, as well as the use of complex actions requiring the coordinated involvement of both hands^14^,^16^. Furthermore, food manipulation complexity in mainly terrestrial species might also be higher as terrestriality affects the availability of food and raw materials to be used as tools^49^,^50^. Terrestrial habitats present a wider range of possible substrates and materials, such as stones and grass stems, in addition to twigs and leaves that can be used as tools^49^,^50^. Similarly, in birds, complex manipulations and tool-use behaviour have been observed in free-ranging species foraging a high proportion of time on the ground, such as ravens and several crow species including New Caledonian crows^51^,^52^.

That terrestrial species showed higher manipulation complexity compared to arboreal ones might also be due to the fact that manipulation and locomotion pose different and frequently opposed selection pressures on primate hand morphology. The forelimb-dominated climbing and suspensory behaviours of arboreal species such as e.g. in gibbons favours a long hand functioning as a grasping hook during suspension and/or climbing that is thus less well suited for manipulative functions^53^,^54^. In contrast, terrestrial quadrupedalism favours a short hand, which is far more compatible with an enhanced thumb/hand relationship, which in turn enables more complex manipulations as in geladas and baboons^55^. However, hand morphology alone does not explain manipulation complexity. The aye-aye (*Daubentonia madagascerensis*), an arboreal species with long digits, especially the thin middle one (D3), would be presumed to have a rather limited capacity for complex manipulations based on its hand morphology. Conversely, in a comparative study, aye-ayes were able to perform more complex manipulations than other lemurs by using the thumbs to secure the food in a sophisticated way^56^. This finding demonstrates that fine motor control in the brain can sometimes override motor limitations imposed by body morphology. It is also consistent with our finding that brain size is related to manipulation complexity, since aye-ayes are very large-brained lemurs.

### The human case

We also found that humans reach by far the highest manipulation complexity, even higher than predicted for our brain size. Human foragers occupy the most complex foraging niche of all mammals^57^, and forager diet requires intensive processing and relies heavily on enhanced manipulative skills^58^. There is ample fossil evidence that, over the course of human evolution, increasingly bipedal habits freed the hand from the constraints of locomotion and hands could evolve primarily for manipulation, including tool use and eventually tool production^59^,^60^. The occurrence of human-like hand proportions and features linked to precision grip in very early hominins even hint at the possibility that manipulative skills were an early autapomorphy of the human lineage that co-evolved with habitual bipedalism and was not necessarily related to stone tool production^61^,^62^,^63^. Admittedly, primate species engaging in tool use also showed higher manipulation complexity than those that do not, suggesting that tool use is also involved in enhancing manipulation complexity. However, as the amount of variation in manipulation complexity explained by tool use or extractive foraging is rather small, brain size and terrestriality may be more important factors.

Our comparative evidence also suggests that terrestriality alone already improves manipulative skills. Thus, in human evolutionary history, the combination of terrestriality with bipedality may have boosted a positive feedback loop with manipulation complexity, far beyond the range of other primates, combined with an unusually large brain and the corresponding cognitive abilities. Together with previous findings that terrestriality is crucial for acquiring and maintaining complex tool variants in primates^16^, our study lends support to the notion that the combination of intelligence and terrestriality may have been a major pacemaker of hominin technological evolution^64^.

That this simple categorization of manipulation complexity yields a consistent pattern of correlation between processing food and brain size obviously does not rule out that finding food may also play an important role in the evolution of cognitive abilities. Although our results add manipulation complexity to a suite of emerging evidence linking cognition with ecological rather than with social factors^1^,^2^,^12^,^13^, the outcomes of the present study are also consistent with a role for social factors, as among primates, the developmental acquisition of all complex manipulative skills has a major social-learning component^65^. However, if social challenges alone (independent of social learning of skills) were responsible for the evolution of the unusually large human brain (e.g.^66^), we would expect human manipulation complexity to be lower than expected for our brain size. The fact that the opposite was actually found, is not favourable to the idea that only purely social challenges were involved.

## Additional Information

**How to cite this article**: Heldstab, S. A. *et al*. Manipulation complexity in primates coevolved with brain size and terrestriality. *Sci. Rep*. **6**, 24528; doi: 10.1038/srep24528 (2016).

## Supplementary Material

Supplementary Information

## Figures and Tables

**Table 1:** PGLS models with manipulation complexity as response variable and tool use, extractive foraging or cognitive test performance as explanatory variables.

Data set	*n*	*λ*	adj. *r*^*2*^	Predictor variable	Estimate	Std. error	*P*-value
excluding *Homo sapiens*	36	0.694	0.125	tool use	0.917	0.374	**0.020**
including *Homo sapiens*	37	0.731	0.172	tool use	1.052	0.361	**0.006**
excluding *Homo sapiens*	36	0.760	0.077	extractive foraging	0.520	0.263	0.056
including *Homo sapiens*	37	0.808	0.081	extractive foraging	0.554	0.271	**0.048**
Deaner *et al*.^20^	15	0	0.552	cog. performance	0.983	0.230	**<0.001**
Reader *et al*.^11^	19	0.444	0.396	cog. performance	0.566	0.158	**0.002**

Significant effects are highlighted in bold face.

**Table 2:** PGLS models with manipulation complexity as response variable and brain size as explanatory variables, terrestriality and diet quality as covariates singly and as combined models (*n* = 34, *Homo sapiens* excluded).

Model	*P*-value model	*λ*	adj*. r*^*2*^	AIC	∆AIC	Predictor variables	Estimate	Std. error	*P*-value
model 1	**<0.001**	0	0.728	**60.773**	–	log brain	1.286	0.387	**0.002**
						log body	−0.551	0.312	0.087
						terrestriality	0.948	0.328	**0.007**
model 2	**<0.001**	0	0.721	62.463	1.690	log brain	1.376	0.429	**0.003**
						log body	−0.618	0.346	0.081
						terrestriality	0.927	0.335	**0.010**
						log diet quality	−0.313	0.607	0.610
model 3	**<0.001**	0	0.663	67.105	6.332	log brain	1.274	0.430	**0.006**
						log body	−0.416	0.343	0.234
model 4	**<0.001**	0	0.659	68.438	7.665	log brain	1.422	0.474	**0.005**
						log body	−0.531	0.376	0.165
						log diet quality	−0.513	0.666	0.447
model 5	**<0.001**	0.147	0.567	68.498	7.725	terrestriality	0.863	0.374	**0.028**
						log body	0.439	0.098	**<0.001**
model 6	**<0.001**	0.211	0.461	73.783	13.010	log diet quality	−0.069	0.659	0.918
						log body	0.531	0.101	**<0.001**

Body mass is always included as covariate.

Significant effects and best-fitting models are highlighted in bold face.

**Table 3:** Mean and width of the 95% confidence intervals obtained through bootstrapping.

Data set		95% CI mean	95% CI width
25 species	nonhuman primates	1.205	1.186–1.225
	*Homo sapiens* included	1.294	above the upper CI
30 species	nonhuman primates	1.236	1.224–1.249
	*Homo sapiens* included	1.294	above the upper CI
