# Supplementary material for: Manipulation complexity in primates coevolved with brain size and terrestriality
Source: Sci Rep. 2016 Apr 14;6:24528. doi: 10.1038/srep24528 (PMC4830942; doi:10.1038/srep24528)
Supplement: Supplementary Information [file srep24528-s1.pdf]

Electronic Supplementary Material to the Manuscript:

"Manipulation complexity in primates coevolved with brain size and terrestriality"

Sandra A. Heldstab, Zaida K. Kosonen, Sonja E. Koski, Judith M. Burkart, Carel P. van Schaik, and Karin Isler

CONTENT

MATERIAL AND METHODS p. 1

Specific brain regions (neocortex and cerebellum) p. 1

Social complexity p. 1

Figure S1. Phylogenetic tree used for the analyses reported in the main text p. 2

Table S1. List of species and data used for this study p. 4

RESULTS p. 7

Supplementary results for the correlates of manipulation complexity p. 7

Results showing that brain size is related to cognitive abilities in our primate sample p. 11

Results of the highest manipulation complexity score ever reached over all bouts p. 12

Results and discussion for the relationship between manipulation complexity and relative neocortex and cerebellum size p. 13

Results and discussion for a potential confounding effect of social complexity p. 14

Results of an alternative coding scheme of diet categories related to demands on manipulative skills p. 15

Results using an alternative phylogenetic tree p. 16

Statistical assumption checks for phylogenetic generalized least-squares p. 17

Notes on the number of observation bouts p. 18

REFERENCES p. 19

Supplementary MATERIAL AND METHODS

Specific brain regions (neocortex and cerebellum)

To test whether manipulation complexity is related to the size of specific brain regions, we examined their correlation with relative neocortex and cerebellum size across primate genera. Some authors argue that relative neocortex size is a better proxy of cognitive ability than whole brain size [1]. Furthermore, relative cerebellum size may be a better proxy for fine motor skills than whole brain size [2], but see [3,4].

Relative neocortex and cerebellum sizes were available for *n*=19 non-human primate genera. Sources of these neuroanatomical measures are given in Supplementary Table S1. The values of neocortex and cerebellum size were $\log_e$ transformed in order to reach residuals evenly distributed around zero.

Social complexity

In this study we do not seek to explain the variation in brain size, but rather variation in manipulation complexity. Nevertheless, the question arises whether sociality may confound the relationship between manipulation complexity and brain size, as suggested by the social complexity hypothesis [5]. Therefore, we additionally tested an alternative model with brain size as response and manipulation complexity as effect and the covariates body mass and foraging group size as proxy for social complexity (data from [6-9], Supplementary Table S1). The values of foraging group size were $\log_e$ transformed in order to reach residuals evenly distributed around zero.

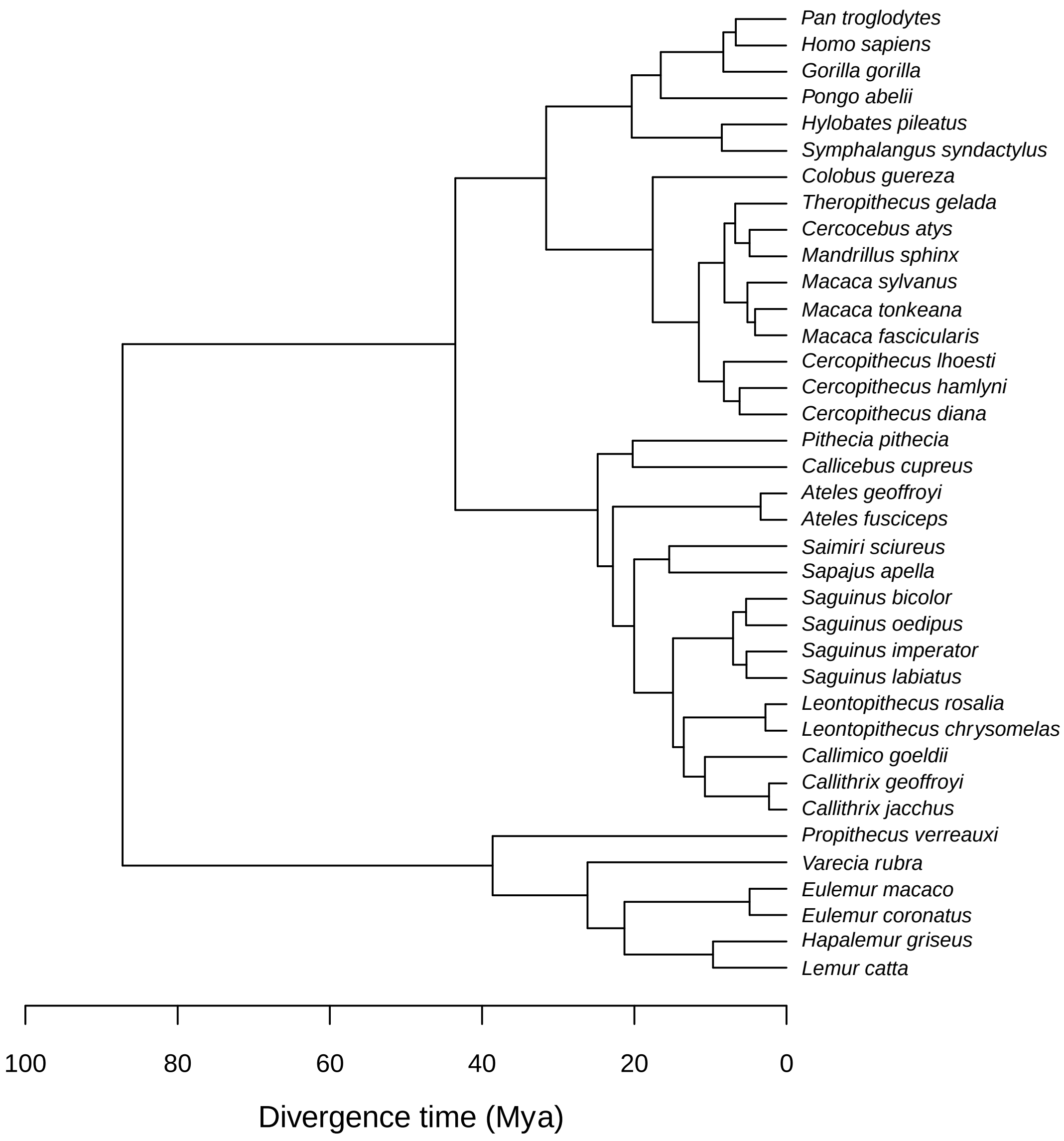

Figure S1. Phylogenetic tree used for the analyses reported in the main text, based on [10].

Tree in Nexus format

#NEXUS

BEGIN TREES;

TRANSLATE

1 Ateles_geoffroyi,

2 Callicebus_cupreus,

3 Callimico_goeldii,

4 Callithrix_geoffroyi,

5 Callithrix_jacchus,

6 Sapajus_apella,

7 Cercocebus_atys,

8 Cercopithecus_diana,

9 Cercopithecus_lhoesti,

10 Colobus_guereza,

11 Eulemur_coronatus,

12 Eulemur_macaco,

13 Gorilla_gorilla,

14 Hapalemur_griseus,

15 Hylobates_pileatus,

16 Lemur_catta,

17 Leontopithecus_rosalia,

18 Macaca_fascicularis,

19 Macaca_sylvanus,

20 Mandrillus_sphinx,

21 Pan_troglodytes,

22 Pithecia_pithecia,

23 Pongo_abelii,

24 Propithecus_verreauxi,

25 Saguinus_bicolor,

26 Saguinus_labiatus,

27 Saguinus_oedipus,

28 Saimiri_sciureus,

29 Symphalangus_syndactylus,

30 Theropithecus_gelada,

31 Varecia_rubra,

32 Ateles_fusciceps,

33 Cercopithecus_hamlyni,

34 Macaca_tonkeana,

35 Homo_sapiens,

36 Leontopithecus_chrysomelas,

37 Saguinus_imperator;

TREE =
(((((16:9.66,14:9.66):11.64,(11:4.86,12:4.86):16.44):4.85,31:26.15):12.45,24:38.6):48.62,((((((((5:2.29,4:2.29):8.41,3:10.7):2.80,(36:2.76,17:2.76):10.74):1.40,((26:5.25,37:5.25):1.75,(27:5.3,25:5.3):1.70):7.9):5.1,(6:15.4,28:15.4):4.6):2.80,(32:3.4,1:3.4):19.40):2.00,(2:20.2,22:20.21):4.60):18.72,(((((8:6.16,33:6.16):2.07,9:8.22):3.29,(((18:4.13,34:4.13):0.99,19:5.12):3.02,((20:4.85,7:4.85):1.88,30:6.73):1.41):3.37):6.07,10:17.58):13.99,((29:8.5,15:8.5):11.82,(23:16.5,(13:8.3,(35:6.6,21:6.55):1.65):8.2):3.82):11.25):11.95):43.7):5.0;

END;

Table S1. List of species and data used for this study.

Species	MC	Study site	# Bouts	# Ind.[$]	ECV [ml][¢]	BoM [g][¢]	Neocortex [g][@]	Cerebellum [g][@]	BoM brain parts [g] [@]	Terr.*	Diet quality	Diet category[&]	Tool use°	Extr. for.[§]	Cog. test[£]	Cog. test**	Group size
Ateles fusciceps	1.35	Parc Zool. et Bot. de Mulhouse	26	4	107.6	9160	-	-	-	0	-	-	0	0	1.28	-0.64	2.75
Ateles geoffroyi	2.48	Zoo Basel	21	7	103.5	7700	70.86	12.44	8000	0	434.2	1	0	0	1.28	-0.64	3.9
Callicebus cupreus	1.85	Zoo Basel	26	4	17.4	887	11.16	1.62	900	0	524.3	1	0	0	-	-	3.4
Callimico goeldii	1.43	Zoo Zurich	23	8	11.1	485	6.48	1.24	480	0	552.5	1	0	0	-	-	6.3
Callithrix geoffroyi	1.32	Zoo Zurich	22	7	9.8	338	4.37	0.78	280	0	541.2	0	0	1	-1.2	-0.86	7.5
Callithrix jacchus	1.33	University of Zurich	24	5	7.4	322	4.37	0.78	280	0	519.1	0	0	1	-1.2	-0.86	8.4
Cercocebus atys	2.24	Bioparco di Roma	21	5	85.9	6200	68.73	10.73	7900	1	439.7	1	0	0	0.25	-0.36	50
Cercopithecus diana	2.03	Parc Zool. et Bot. de Mulhouse	31	3/2	57.3	3900	47.55	6.29	4850	0	554.2	1	0	0	0.39	0.14	22
Cercopithecus hamlyni	3.84	Parc Zool. et Bot. de Mulhouse	25	5	51.2	2097	47.55	6.29	4850	0.5	605.2	0	0	0	0.39	0.14	8
Cercopithecus lhoesti	2.77	Parc Zool. et Bot. de Mulhouse	22	5	66.5	3450	47.55	6.29	4850	0.5	362.2	0	0	1	0.39	0.14	30
Colobus guereza	2.38	Toni’s Zoo Rothenburg	21	3	72.6	7503	50.91	8.65	7000	0	348.3	0	0	0	-	-0.64	9.3
Eulemur coronatus	1.60	Parc Zool. et Bot. de Mulhouse	20	5	19.2	1422	12.21	3.33	1400	0	493.4	1	0	0	-0.5	-0.71	5.5
Eulemur macaco	1.23	Parc Zool. et Bot. de Mulhouse	22	2/2/2	22.7	1908	12.21	3.33	1400	0	453.6	1	0	0	-0.5	-0.71	8.5
Gorilla gorilla	5.00	Zoo Zurich	33	9	434.4	71500	341.44	69.25	105000	1	359.0	0	0	1	0.96	0.87	10.5
Hapalemur griseus	1.78	Parc Zool. et Bot. de Mulhouse	27	2/1	13.7	935	-	-	-	0	296.0	0	0	0	-	-	4.4

Species	MC	Study site	# Bouts	# Ind.[$]	ECV [ml][¢]	BoM [g][¢]	Neocortex [g][@]	Cerebellum [g][@]	BoM brain parts [g] [@]	Terr.*	Diet quality	Diet category[&]	Tool use[°]	Extr. for.[§]	Cog. test[£]	Cog. test**	Group size
Homo sapiens	5.40	University of Zurich	30	12	1212.7	56700	1006.53	137.42	65000	1	690.6	1	1	1	-	-	37.7
Hylobates pileatus	2.14	Zoo Zurich	21	5/4	90.5	5440	65.8	12.08	5700	0	486.5	1	0	0	0.11	-0.79	4
Lemur catta	1.48	Zoo Basel	21	4	23.4	2210	10.69	2.59	-	0.5	527.6	1	0	0	-0.8	-0.79	14
Leontopithecus chrysomelas	1.63	University of Zurich	30	6	11.8	655	-	-	-	0.5	489.8	1	0	1	-	-1.36	4.75
Leontopithecus rosalia	1.20	Zoo Zurich	20	1	12.6	594.5	-	-	-	0	471.2	1	0	1	-	-1.36	5.4
Macaca fascicularis	4.11	Zoo Basel	27	17	61.0	3516	63.48	8.97	7800	0.5	432.4	1	1	1	0.55	1.64	27
Macaca sylvanus	3.76	Toni's Zoo Rothenburg	21	9	94.8	9625	63.48	8.97	7800	1	429.5	1	0	1	0.55	1.64	24
Macaca tonkeana	3.00	Parc Zool. et Bot. de Mulhouse	24	3	93.7	9000	63.48	8.97	7800	0.5	471.8	1	0	0	0.55	1.64	24
Mandrillus sphinx	2.21	Bioparco di Roma	38	12	137.3	12800	95.75	8.74	-	1	482.2	1	0	0	0.43	-0.57	215
Pan troglodytes	4.38	Zoo Basel	26	10	391.6	33700	291.59	43.66	46000	0.5	491.5	1	1	1	1.66	2.80	5.6
Pithecia pithecia	1.86	Zoo Basel	37	7	31.6	1816	21.03	3.91	1500	0	479.9	1	0	0	-	-	4.4
Pongo abelii	3.89	Zoo Zurich	35	7	349.7	41151	200.26	97.80	73500	0	581.2	1	1	1	1.75	1.71	1.7
Propithecus verreauxi	1.44	Parc Zool. et Bot. de Mulhouse	27	1/1	26.1	3250	13.17	3.96	3480	0	318.8	0	0	0	-	-1.00	5.5
Saguinus bicolor	1.00	Parc Zool. et Bot. de Mulhouse	21	2/2/5	9.5	473	5.89	0.98	380	0	500.0	1	0	0	-	0.43	4.9
Saguinus imperator	1.62	Zoo Zurich	21	2	10.7	446	5.89	0.98	380	0	-	-	0	1	-	0.43	4

Species	MC	Study site	# Bouts	# Ind.$	ECV [ml]¢	BoM [g]¢	Neocortex [g]@	Cerebellum [g]@	BoM brain parts [g] @	Terr.*	Diet quality	Diet category&	Tool use°	Extr. for.§	Cog. test £	Cog. test**	Group size
Saguinus labiatus	2.27	Zoo Basel	22	2	10.0	520	5.89	0.98	380	0	493.0	1	0	0	-	0.43	5
Saguinus oedipus	1.70	Zoo Basel	37	4	9.7	427	5.89	0.98	380	0	684.4	1	0	0	-	0.43	5.6
Saimiri sciureus	1.94	University of Zurich	31	7	23.5	821	15.54	2.26	660	0	435.4	1	0	1	-0.9	-0.79	45
Sapajus apella	2.54	Zoo Zurich	41	6	64.2	2501	46.43	7.87	3100	0	543.0	1	1	1	0.19	1.43	13.9
Symphalangus syndactylus	3.08	Zoo Zurich	24	3	124.5	11295	-	-	-	0	432.0	0	0	0	-	-	3.6
Theropithecus gelada	4.17	Zoo Zurich	23	27	123.2	14171	-	-	-	1	313.2	0	0	0	-	-0.43	103.8
Varecia rubra	1.33	Parc Zool. et Bot. de Mulhouse	21	4	29.4	3300	15.29	4.29	3000	0	454.0	1	0	0	-0.4	-	5.5

Notes:

MC = manipulation complexity means, ECV = female endocranial volume, BoM = female body mass,
# Bouts = number of observation bouts, # Ind. = Number of individuals observed

$Multiple numbers of individuals indicate that several groups from the same species were observed.

¢All brain and body mass measurements were from Lonsdorf and Ross [11] and van Woerden et al. [12,13] except the body mass for *Saguinus imperator* was taken from Rowe and Myers [7].

@Neocortex and cerebellum size and associated body mass measurements were taken from [14-16].

*Terrestriality, 1 = terrestrial (>60%), 0.5 = semi-terrestrial (>20%), 0 = arboreal

&Diet category, 1 = fruits and insects, 0 = gum and leaves

°Tool use, 0 = non-tool using species, 1 = tool-using species

§Extractive foraging, 0 = non-extractive foraging species, 1 = extractive foraging species

£Cognitive test performance measured by [17]

**Cognitive test performance measured by [18]

Supplementary RESULTS

Supplementary results for the correlates of manipulation complexity

Manipulation complexity is significantly higher in primate species that regularly use tools and nearly significantly higher in species that exhibit extractive foraging (Supplementary Fig. S2).

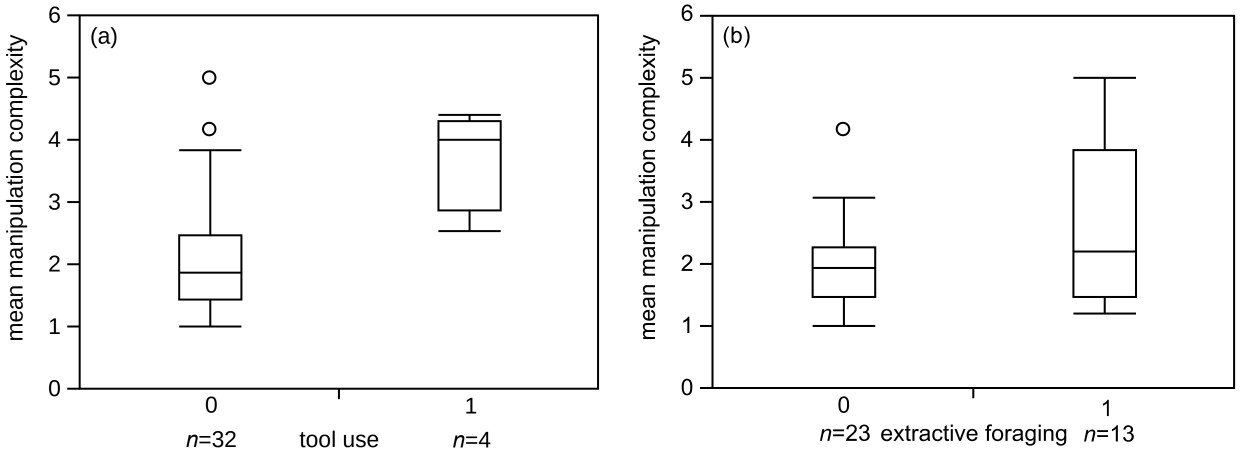

Figure S2. Manipulation complexity (a) in primate species that regularly use tools (*P*=0.020) and (b) in species that exhibit extractive foraging (*P*=0.056) (*n*=36 primate species, *Homo sapiens* excluded). Statistical details see main text.

Manipulation complexity is correlated not only with relative brain size, but also with cognitive test performance across primate genera (Supplementary Fig. S3). There is no correlation with diet quality (Supplementary Fig. S3).

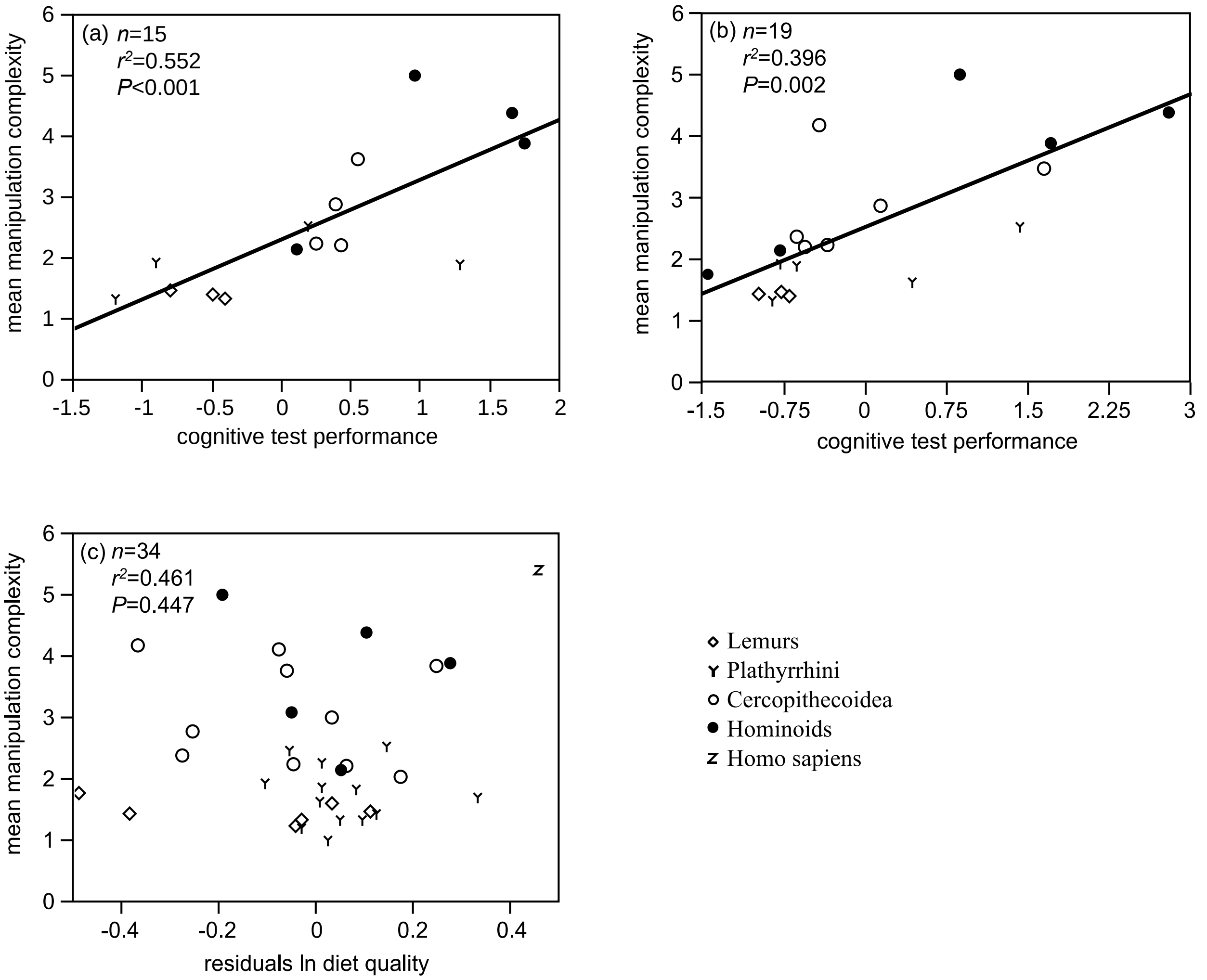

Figure S3. (a) Relationship between manipulation complexity and cognitive test performance (raw genus values measured by [17]). Statistical details of the PGLS regression models see Table 1 in the main text. (b) Relationship between manipulation complexity and cognitive test performance (raw genus values measured by [18]). Statistical details of the PGLS regression models see Table 1 in the main text. (c) Diet quality (controlled for body mass) and manipulation complexity are not correlated (raw species values). Statistical details of the PGLS regression models see Table 2 in the main text. The symbols denote different primate taxa, for exact species values see Supplementary Table S1.

Alternative models, including the interaction terms between brain size and terrestriality and brain size and diet quality, show that the effect of neither interaction is statistically significant (Supplementary Table S2).

Table S2. PGLS models with manipulation complexity as response variable and brain size as explanatory variables, terrestriality and diet quality as covariates singly and as interaction effects with brain size (n=34, excluding *Homo sapiens*). Including body mass as covariate.

***P*-value model**	**λ**	**adj. r^2**	**AIC**	**ΔAIC**	**predictor variables**	**estimate**	**std. error**	***P*-value**
**<0.001**	0	0.726	61.860	-	log brain	1.175	0.319	**0.003**
					log body	−0.580	0.315	0.076
					terrestriality	0.261	1.400	0.853
					log brain * terrestriality	-0.267	0.301	0.382
**<0.001**	0	0.683	66.841	4.981	log brain	6.855	3.054	**0.033**
					log body	−0.582	0.375	0.132
					log diet quality	2.691	1.894	0.166
					log brain * log diet quality	−0.877	0.487	0.082

Significant effects and best-fitting models are highlighted in bold face.

The results of PGLS models that include *Homo sapiens* (Supplementary Table S3) are largely similar to those that exclude *Homo sapiens* (Table 2, main text).

Table S3. PGLS models with manipulation complexity as response variable and brain size as explanatory variables, terrestriality and diet quality as covariates singly and as combined models (n=35, including *Homo sapiens*). Including body mass as covariate.

model	*P*-value model	λ	adj. r^2	AIC	ΔAIC	predictor variables	estimate	std. error	*P*-value
**model 1**	**<0.001**	0	0.778	**61.623**	-	log brain	1.175	0.319	**<0.001**
						log body	−0.471	0.268	0.089
						terrestriality	0.919	0.320	**0.007**
model 2	**<0.001**	0	0.774	63.181	1.558	log brain	1.306	0.386	**0.002**
						log body	−0.584	0.326	0.084
						terrestriality	0.902	0.324	**0.009**
						log diet quality	−0.362	0.586	0.542
model 3	**<0.001**	0	0.728	67.888	6.265	log brain	1.267	0.352	**0.001**
						log body	−0.411	0.296	0.175
model 4	**<0.001**	0	0.725	69.215	7.592	log brain	1.445	0.422	**0.002**
						log body	−0.568	0.360	0.125
						log diet quality	−0.500	0.644	0.444
model 5	**<0.001**	0.157	0.621	71.351	9.728	terrestriality	0.976	0.370	**0.013**
						log body mass	0.460	0.098	**<0.001**
model 6	**<0.001**	0.148	0.551	77.681	16.058	log diet quality	0.455	0.615	0.465
						log body	0.607	0.092	**<0.001**

Significant effects and best-fitting models are highlighted in bold face.

Results showing that brain size is related to cognitive abilities in our primate sample

Deaner et al 2007[17] and Reader et al 2011[18] showed with large data sets that cognitive abilities are related to brain size in primates. Consistent with these findings also in our study cognitive abilities and brain size are positively linked (Supplementary Table S4).

Table S4. PGLS models with cognitive abilities measured either by Deaner et al 2007[17] (n=15) or Reader et al 2011[18] (n=19) as response variable and brain size and body mass as explanatory variables.

data set	*P*-value model	λ	adj. r^2	predictor variables	estimate	std. error	*P*-value
Deaner et al 2007[17]	**<0.001**	0	0.840	log brain	0.837	0.341	**0.030**
				log body	-0.089	0.270	0.747
Reader et al 2011[18]	**0.010**	0	0.371	log brain	1.681	0.786	**0.048**
				log body	-0.889	0.622	0.172

Significant effects are highlighted in bold face.

Results of the highest manipulation complexity score ever reached over all bouts

We conducted additional tests with the highest manipulation complexity score ever reached by a species over all bouts and its relationship to brain size, terrestriality and diet quality. The results of PGLS regression models using this manipulation complexity scoring are reported in Supplementary Table S5. Although *P*-values vary slightly in comparison with Tables 2, on the whole the results are very similar (Supplementary Table S5).

Table S5. PGLS models with the highest manipulation complexity score reached over all bouts as response variable and brain size as explanatory variables, terrestriality and diet category as covariates singly and as combined models (*n*=34, *Homo sapiens* excluded). Including body mass as covariate.

model	*P*-value model	*λ*	adj. r^2	AIC	ΔAIC	predictor variables	estimate	std. error	*P*-value
**model 1**	**<0.001**	0	0.611	**104.737**	-	log brain	1.870	0.738	**0.017**
						log body	−0.952	0.595	0.120
						terrestriality	2.132	0.627	**0.002**
model 2	**<0.001**	0	0.598	106.686	1.949	log brain	1.800	0.822	**0.037**
						log body	−0.889	0.675	0.198
						terrestriality	2.148	0.642	**0.002**
						diet category	0.242	1.163	0.836
model 3	**<0.001**	0.113	0.467	108.907	4.170	terrestriality	1.933	0.677	**0.008**
						log body	0.488	0.174	**0.009**
model 4	**<0.001**	0	0.478	113.824	9.087	log brain	1.845	0.855	**0.039**
						log body	−0.648	0.681	0.349
model 5	**<0.001**	0	0.461	115.792	11.055	log brain	1.909	0.950	**0.054**
						log body	−0.708	0.779	0.371
						diet category	−0.223	1.336	0.869
model 6	**<0.001**	0.305	0.234	115.981	11.244	diet category	0.374	1.214	0.760
						log body	0.677	0.198	**0.002**

Significant effects, trends and best-fitting models are highlighted in bold face.

Results and discussion for the relationship between manipulation complexity and relative neocortex and cerebellum size

This section reports additional results on the relationship between the size of specific brain regions (neocortex or cerebellum size) and manipulation complexity (Supplementary Table S6). A positive correlation between manipulation complexity and relative cerebellum size was not found in any model. Relative neocortex size on the other hand was always positively correlated with manipulation complexity. This may indicate a closer link between manipulation complexity and cognitive rather than motor skills. However, the cerebellum is involved not only in sensory-motor control and automatized learning of motor skills, but may also play a role in understanding and producing complex behavioural sequences including tool use [3,4]. Our results on brain parts, depending on a relatively small sample, must therefore be regarded with caution.

Table S6. PGLS models with manipulation complexity as response variable and neocortex / cerebellum size and body mass as explanatory variables.

data set	*n*	***P*-value model**	**λ**	**adj. r^2**	**AIC**	**ΔAIC**	**predictor variables**	**estimate**	**std. error**	***P*-value**
excluding *H. sapiens*	19	**<0.001**	0	0.786	**30.132**	-	log neocortex	0.730	0.327	**0.040**
							log body	0.016	0.249	0.950
excluding *H. sapiens*	19	**<0.001**	0	0.791	30.440	0.308	log neocortex	0.960	0.377	**0.023**
							log cerebellum	-0.660	0.559	0.256
							log body	0.366	0.385	0.357
excluding *H. sapiens*	19	**<0.001**	0.468	0.669	33.322	3.190	log cerebellum	-0.323	0.512	0.537
							log body	0.796	0.416	0.073
including *H. sapiens*	20	**<0.001**	0	0.845	**30.857**	-	log neocortex	0.857	0.252	**0.003**
							log body	-0.065	0.209	0.761
including *H. sapiens*	20	**<0.001**	0	0.844	31.769	0.912	log neocortex	1.088	0.351	**0.007**
							log cerebellum	-0.504	0.532	0.358
							log body	0.167	0.323	0.612
including *H. sapiens*	20	**<0.001**	0.230	0.722	38.859	8.002	log cerebellum	0.498	0.453	0.287
							log body	0.192	0.384	0.624

Significant effects are highlighted in bold face.

Results and discussion for a potential confounding effect of social complexity

This section reports additional results of an alternative model testing whether sociality confounds the relationship between brain size and manipulation complexity (Supplementary Table S7). Foraging group size of each species was used as a proxy for social complexity (listed in Supplementary Table S1). The relationship between brain size and manipulation complexity persists even after controlling for foraging group size. Our results are therefore broadly consistent with the idea that social factors such as group size may not be the only important feature of primate brain size evolution; selection on ecological factors such as foraging skills may have been important too [4,19,20]. However, the outcomes of the present study do not rule out some influence of social factors, as among primates the developmental acquisition of all complex manipulative skills has a major social-learning component [21], which could well be affected by group size and composition, as well as the degree of social tolerance.

Regarding this analysis, we must caution this sample is not optimal to identify the variables affecting brain size variation in primates. The aim of the current study was not to conduct such an analysis. We therefore do not claim that variation in manipulation complexity is the sole or most important variable influencing brain size evolution.

Table S7. PGLS models with brain size as response variable, manipulation complexity as explanatory variables and foraging group size as potential confounding effect (*n*=37, *Homo sapiens* included). Including body mass as covariate.

***P*-value model**	**λ**	**adj. r^2**	**predictor variables**	**estimate**	**std. error**	***P*-value**
			manipulation complexity	0.137	0.057	**0.022**
**<0.001**	0.111	0.928	log body	0.719	0.046	**<0.001**
			log foraging group size	0.053	0.044	0.235

Significant effects are highlighted in bold face.

Results of an alternative coding scheme of diet categories related to demands on manipulative skills

To investigate whether the results reported in this study are robust with respect to different coding schemes of the influence of diet on manipulation complexity, we conducted analogous tests with diet categories related to demands on manipulative skills instead of continuous estimates of diet quality. The results of PGLS regression models using this scheme are reported in Supplementary Table S8. Although *P*-values vary slightly in comparison with Tables 2 and Supplementary Table S3, on the whole the results are very similar.

Table S8. PGLS models with manipulation complexity as response variable and brain size as explanatory variables, terrestriality and diet category as covariates singly and as combined models (*n*=34, *Homo sapiens* excluded). Including body mass as covariate.

model	*P*-value model	λ	adj. r^2	AIC	ΔAIC	predictor variables	estimate	std. error	*P*-value
**model 1**	**<0.001**	0	0.745	**59.345**	-	log brain	1.354	0.376	**0.001**
						log body	−0.608	0.303	0.054
						terrestriality	0.900	0.319	**0.008**
						diet category	−0.362	0.206	0.090
model 2	**<0.001**	0	0.728	60.773	1.428	log brain	1.286	0.387	**0.002**
						log body	−0.551	0.312	0.087
						terrestriality	0.948	0.328	**0.007**
model 3	**<0.001**	0	0.686	65.609	6.264	log brain	1.353	0.418	**0.003**
						log body	−0.489	0.333	0.153
						diet category	−0.412	0.228	0.082
model 4	**<0.001**	0	0.663	67.105	7.760	log brain	1.274	0.430	**0.006**
						log body	−0.416	0.343	0.234
model 5	**<0.001**	0.147	0.567	68.498	9.153	terrestriality	0.863	0.374	**0.028**
						log body	0.439	0.098	**<0.001**
model 6	**<0.001**	0.243	0.483	71.436	12.091	diet category	−0.361	0.240	0.143
						log body	0.528	0.096	**<0.001**

Significant effects and best-fitting models are highlighted in bold face.

Results using an alternative phylogenetic tree

To investigate whether the results reported in this study are robust with respect to different tree phylogenies, we conducted analogous tests using the 10k trees phylogeny [22]. The results of PGLS regression models using this phylogeny are reported in Supplementary Table S9. The positive correlation between manipulation complexity and relative brain size remains unaffected by the type of phylogeny that is used which corroborates the stability of our results. Furthermore, also with the 10k trees phylogeny manipulation complexity is best explained by brain size and terrestriality.

Table S9. PGLS models with manipulation complexity as response variable and brain size as explanatory variables, terrestriality and diet quality as covariates singly and as combined *models (n*=33, *Ateles fusciceps, Saguinus labiatus, Saguinus imperator* and *Homo sapiens* excluded). As *Callicebus cupreus* is not present in the 10k tree phylogeny but the only species of that genus in our analyses, we replaced it by a sister species (*Callicebus moloch*). Including body mass as covariate.

model	*P*-value model	*λ*	adj. r^2	AIC	ΔAIC	predictor variables	estimate	std. error	*P*-value
**model 1**	**<0.001**	0	0.760	**55.990**	-	log brain	1.342	0.370	**0.001**
						log body	−0.568	0.298	0.066
						terrestriality	0.948	0.328	**0.007**
model 2	**<0.001**	0	0.754	57.588	1.598	log brain	1.439	0.410	**0.002**
						log body	−0.655	0.336	0.061
						terrestriality	0.930	0.319	**0.007**
						log diet quality	−0.339	0.579	0.563
model 3	**<0.001**	0	0.694	63.119	7.129	log brain	1.330	0.418	**0.003**
						log body	−0.432	0.332	0.203
model 4	**<0.001**	0	0.690	64.330	8.340	log brain	1.486	0.460	**0.003**
						log body	−0.577	0.376	0.136
						log diet quality	−0.540	0.644	0.409
model 5	**<0.001**	0.155	0.570	65.590	9.600	terrestriality	0.869	0.369	**0.025**
						log body	0.454	0.100	**<0.001**
model 6	**<0.001**	0.237	0.446	71.079	15.089	log diet quality	−0.046	0.650	0.944
						log body	0.539	0.106	**<0.001**

Significant effects and best-fitting models are highlighted in bold face.

Statistical assumption checks for phylogenetic generalized least-squares

Based on the findings by Matthews et al. [23], ordinal ranked data, such as manipulation complexity measured in this study, can be treated as pseudo-continuous for PGLS analyses. The following assumption checks of Phylogenetic generalized least squares, described by Mundry [24], were tested:

- In order to reach evenly distributed residuals around zero, all continuous variables were log-transformed.
- Categorical predictors (terrestriality and diet category) did not have 'too rare levels' (each level was present at least five times).
- Absence of strong collinearity among the predictors.
- Homogeneity and normality of the distribution of the residuals (and the response) was visually inspected.
- Absence of influential cases: No severely influential outliers were detected.

Notes on the number of observation bouts

To investigate whether the results in this study are robust with respect to different number of bouts observed for each species, we conducted all tests with a sample reduced to a fixed number of the first 20 observation bouts per species. As the results were largely identical to the total sample reported here, the reduced sample results are not reported here. Furthermore, we investigated whether a minimum of 20 bouts per species is enough to see the potential of manipulation complexity per species. We did this by constructing so-called collector's (saturation) curves per species and examining after how many observation bouts the highest manipulation complexity category was reached (Supplementary Fig. S4). As the highest manipulation complexity category was reached within 20 bouts for most of the observed species for which we had observed 25 bouts or more (15 out of 17 species, except for *Mandrillus sphinx* and *Pithecia pithecia*), we conclude that our results are robust against changes in observation time.

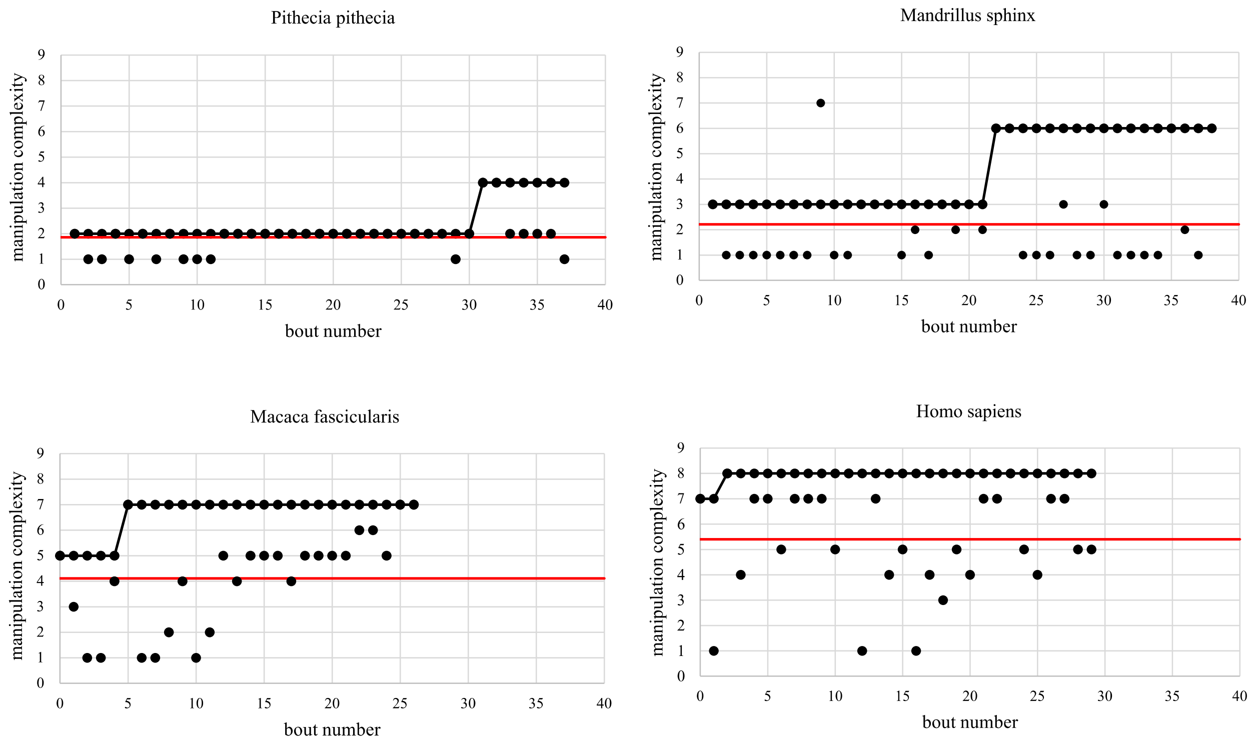

Figure S4. Saturation curves per species for examining after how many observation bouts the highest manipulation complexity category was reached. The red line indicates the mean manipulation complexity of a particular species.

REFERENCES

1 Byrne, R. W. & Corp, N. Neocortex size predicts deception rate in primates. *Phil Trans R Soc B* **271**, 1693-1699 (2004).

2 Day, L. B., Westcott, D. A. & Olster, D. H. Evolution of bower complexity and cerebellum size in bowerbirds. *Brain Behav. Evol.* **66**, 62-72 (2005).

3 Barton, R. A. & Venditti, C. Rapid evolution of the cerebellum in humans and other great apes. *Curr. Biol.* **24**, 2440-2444 (2014).

4 Barton, R. A. Embodied cognitive evolution and the cerebellum. *Phil. Trans. R. Soc. B* **367**, 2097-2107 (2012).

5 Byrne, R. W. & Whiten, A. *Machiavellian Intelligence. Social Expertise and the Evolution of Intellect in Monkeys, Apes, and Humans*. (Clarendon Press, 1988).

6 Willems, E. P. & van Schaik, C. P. Collective action and the intensity of between-group competition in nonhuman primates. *Behav. Ecol.* **26**, 625-631 (2015).

7 Rowe, N. & Myers, M. *All the World's Primates*, (2011) Available at: http://www.alltheworldsprimates.org>, (Date of access: 08/09/2014).

8 Myers, P. *et al. The Animal Diversity Web*.(2006) Available at: *http://animaldiversity.ummz.umich.edu/*, (Date of access: 05/09/2014).

9 Willems, E. P., Hellriegel, B. & van Schaik, C. P. The collective action problem in primate territory economics. *Proc. R. Soc. B* **280**, 20130081 (2013).

10 Perelman, P. *et al.* A molecular phylogeny of living primates. *PLoS Gen.* **7**, e1001342 (2011).

11 Lonsdorf, E. V. & Ross, S. R. in *The Evolution of Primate Societies* (eds John C. Mitani *et al.*) Ch. 11, 245-268 (University of Chicago Press, 2012).

12 van Woerden, J. T., van Schaik, C. P. & Isler, K. Effects of seasonality on brain size evolution: Evidence from strepsirrhine primates. *Am. Naturalist* **176**, 758-767 (2010).

13 van Woerden, J. T., Willems, E. P., van Schaik, C. P. & Isler, K. Large brains buffer energetic effects of seasonal habitats in catarrhine primates. *Evolution* **66**, 191-199 (2012).

14 Stephan, H., Frahm, H. & Baron, G. New and revised data on volumes of brain structure in insectivores and primates. *Folia Primatol.* **35**, 1-29 (1981).

15 Rilling, J. K. & Insel, T. R. The primate neocortex in comparative perspective using magnetic resonance imaging. *J. Hum. Evol.* **37**, 191-223 (1999).

16 Bush, E. C. & Allman, J. M. The scaling of frontal cortex in primates and carnivores. *Proc. Natl. Acad. Sci.* **101**, 3962-3966 (2004).

17 Deaner, R. O., Isler, K., Burkart, J. & van Schaik, C. Overall brain size, and not encephalization quotient, best predicts cognitive ability across non-human primates. *Brain Behav. Evol.* **70**, 115-124 (2007).

18 Reader, S. M., Hager, Y. & Laland, K. N. The evolution of primate general and cultural intelligence. *Phil. Trans. R. Soc. B* **366**, 1017-1027 (2011).

19 Byrne, R. W. *The Technical Intelligence hypothesis: An Additional Evolutionary Stimulus to Intelligence?* , 289-311 (Cambridge University Press, 1997).

20 Parker, S. T. & Gibson, K. R. Object manipulation, tool use and sensorimotor intelligence as feeding adaptations in *Cebus* monkeys and great apes. *J. Hum. Evol.* **6**, 623-641 (1977).

21 van Schaik, C. P. & Pradhan, G. R. A model for tool-use traditions in primates: implications for the coevolution of culture and cognition. *J. Hum. Evol.* **44**, 645-664 (2003).

22 Arnold, C., Matthews, L. J. & Nunn, C. L. The 10kTrees website: a new online resource for primate phylogeny. *Evol. Anthropol.* **19**, 114-118 (2010).

23 Matthews, L. J., Arnold, C., Machanda, Z. & Nunn, C. L. Primate extinction risk and historical patterns of speciation and extinction in relation to body mass. *Proc. Natl. Acad. Sci.* **278**, 1256-1263 (2010).

24 Mundry, R. in *Modern Phylogenetic Comparative Methods and Their Application in Evolutionary Biology* (ed László Zsolt Garamszegi) 131-153 (Springer, 2014).
